# Pleural Epithelioid Hemangioendothelioma: An Ultrarare Cause for Recurrent Pleural Effusion Managed With Trametinib and Pazopanib

**DOI:** 10.7759/cureus.60002

**Published:** 2024-05-09

**Authors:** Ali Khreisat, Paul Bozyk, Gloria Hong, Casey P Schukow

**Affiliations:** 1 Internal Medicine, Corewell Health William Beaumont University Hospital, Royal Oak, USA; 2 Pulmonary and Critical Care Medicine, Corewell Health William Beaumont University Hospital, Royal Oak, USA; 3 Pathology, Corewell Health William Beaumont University Hospital, Royal Oak, USA

**Keywords:** video-assisted thoracoscopic surgery (vats), trapped lung, pleural effusion, recurrent epithelioid hemangioendothelioma, pleural epithelioid hemangioendothelioma

## Abstract

Epithelioid hemangioendothelioma (EHE) is an extremely rare sarcoma of vascular origin. Primary pleural involvement is extremely under-reported and tends to have a more aggressive course. We report a case of pleural EHE in a Caucasian female in her 50s with a two-month history of dyspnea and chest pain. Investigations, including video-assisted thoracoscopy, revealed extensive pleural scarring and inflammation. Management with trametinib and pazopanib led to a stable disease course, reduction in the frequency of pleural effusion recurrence, and improvement in cancer-related pain.

## Introduction

Epithelioid hemangioendothelioma (EHE) is a low-to-intermediate-grade vascular malignancy that can be locally aggressive [1]. This tumor is extremely rare with an approximate incidence of 0.038/100,000 per year and a prevalence of <1/1,000,000, peaking in the fourth to fifth decade of life [2]. Although EHE may occur anywhere in the body, it is most commonly present as a solitary soft tissue mass arising most frequently from a blood vessel (∼50% of cases). However, metastatic disease to the lung, liver, or bone occurs in at least 50% of patients [2]. EHE arising from the pleura is uncommon and under-reported but has been described to be more aggressive than EHE involving other sites [3]. We present a unique case of pleural EHE presenting with recurrent pleural effusion, severe pleuritis, scarring, and trapped lung syndrome.

## Case presentation

**Figure 1:**
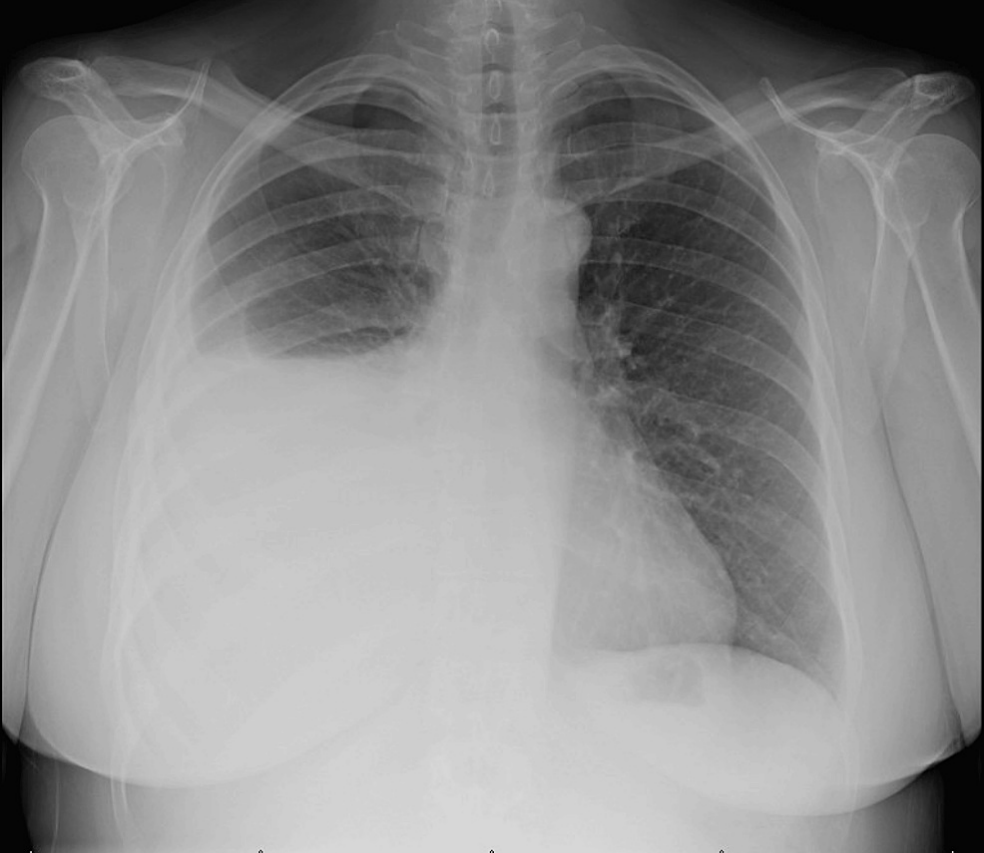
The patient's chest X-ray showing moderate to large right pleural effusion. The cardiomediastinal silhouette is within normal limits. No overt pulmonary congestion.

A Caucasian female in her 50s with no significant medical history was referred to the pulmonary clinic for two months of progressive exertional dyspnea, fatigue, persistent right pleuritic chest pain, and 20 pounds of unintentional weight loss. She denied fever, night sweats, prior cigarette smoking, or asbestos exposure. Physical examination showed normal vital signs, diminished breath sounds over the right lower lung and new fingernail clubbing. Chest x-ray showed a large right pleural effusion (Figure 1).

**Figure 2:**
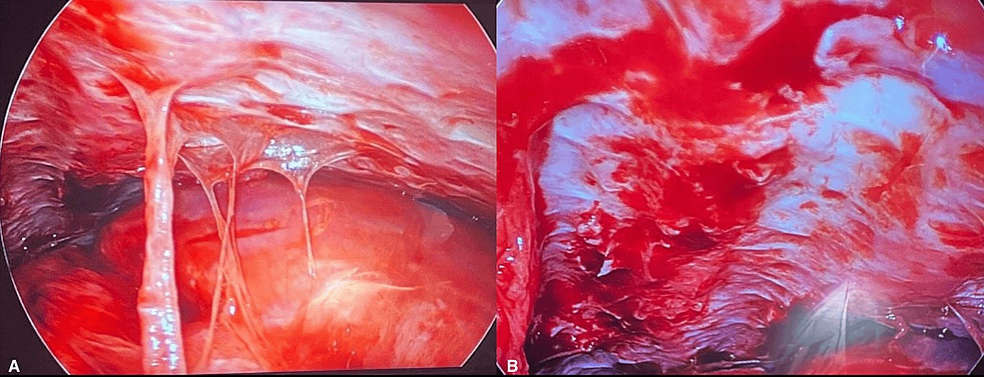
Video-assisted thoracoscopy showing chronically inflamed right parietal pleura with marked pleural thickening and fibrosis (as shown in A and B). The right lower lobe of the lung also had a thick fibrous scar partially entrapping it (A).

Thoracentesis identified exudative lymphocyte-predominant pleural fluid with normal pH, high protein, high lactate dehydrogenase (LDH), normal glucose, negative infectious workup, along with fluid cytology negative for malignancy (Table 1). Chest computed tomography scan showed pleural calcifications, nodular thickening of the pleura, and a 1.8 cm right paraoesophageal lymph node. She underwent esophagogastroduodenoscopy (EGD) with endoscopic ultrasound-guided lymph node biopsy that was unrevealing. In two months, the patient underwent five thoracenteses with negative cytology. To address the recurrent pleural effusions, she underwent video-assisted thoracoscopic surgery, which showed chronically inflamed right parietal pleura with pleural thickening, fibrosis and partially entrapped right lower lung lobe by a thick fibrous scar (Figure 2). Multiple biopsies of the parietal pleura using a mediastinoscopy biopsy forceps were sent to pathology.

**Table 1:** The patient's pleural fluid studies after initial thoracentesis showing exudative lymphocyte-predominant pleural effusion

Pleural fluid analysis		Reference range
Appearance	Turbid serosanguineous	-
Total protein	4.6 g/dl	< 1.5 g/dl
pH	7.44	7.60-7.64
RBC	<2000/mcl	2-1000/mcl
WBC	2800/mcl	< 1000/mcl
Neutrophils %	2%	-
Lymphocytes %	95%	-
Monocyte %	3%	-
Glucose	92 mg/dl	>60 mg/dl
Lactate dehydrogenase	323 U/L	140-280 U/L
Adenosine deaminase	9 U/L	0-30 U/L
Cholesterol	85 mg/dl	<45 mg/dl

**Figure 3:**
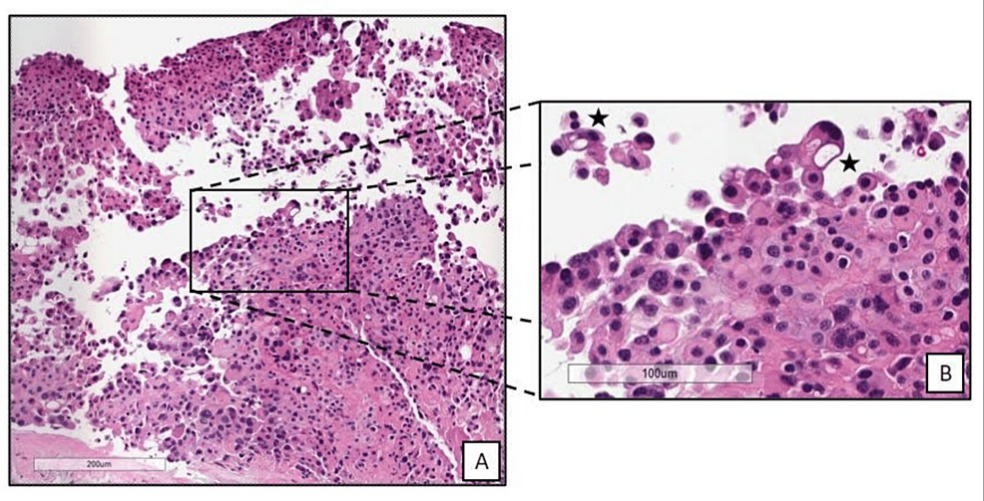
Hematoxylin and eosin stain of epithelioid hemangioendothelioma (EHE). At low power (200 um, A), a broken-apart nest of plump, epithelioid neoplastic cells with densely eosinophilic cytoplasm is appreciated. At higher power (100 um, right, B), prominent nuclear and cytoplasmic pleomorphism is noted, with some cells having intracytoplasmic vacuoles (“blister cells” see stars).

**Figure 4:**
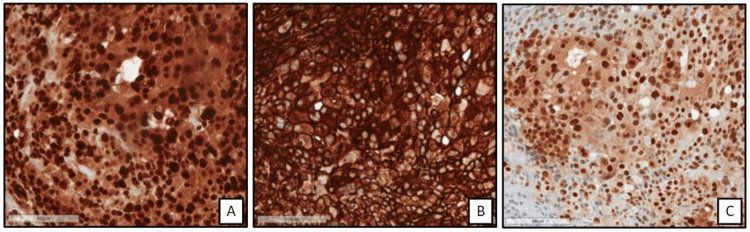
Immunohistochemistry of tissue biopsy. Strong nuclear staining of ERG (left, A) and cytoplasmic/membranous staining of CD31 (middle, B) supports vascular etiology of this neoplasm. CAMTA1 nuclear positivity (right, C) is both sensitive and specific for CAMTA1 fusion-driven epithelioid hemangioendothelioma (EHE), as seen in approximately 90% of cases. CD: Cluster of Differentiation CAMTA1: Calmodulin-Binding Transcription Activator 1

The pleural biopsy showed plump neoplastic epithelioid cells with densely eosinophilic cytoplasm organized in nests on low power (Figure 3A). At high power (Figure 3B), these cells have prominent nuclear and cytologic pleomorphism, with many cells forming intracytoplasmic vacuoles. The differential diagnosis is broad, and the immunophenotyping of these neoplastic cells was negative or nondiagnostic for multiple lineages, including lung (CK-7, TTF-1), colorectal (SATB-2), epithelial (CK-5, AE-1/3, Claudin-4, MOC-31), mesothelial (calretinin, WT-1, D2-40, retained BAP-1), mesenchymal (desmin), smooth muscle (SMA), skeletal muscle (Myogenin, MyoD1), melanocytic (SOX-10, MART-1), hepatocellular (HepPar-1, Arginase), sex cord-stromal (inhibin, HCG). STAT6, a sensitive and specific marker for solitary fibrous tumor (SFT), was also negative. Notably, two vascular markers (ERG, CD31) were both diffusely positive in these neoplastic cells, while a third vascular marker (CD31) was negative. This immune profile, given the patient’s clinical context and cell morphology, raised suspicion for EHE, which was confirmed by positive immunostaining for CAMTA1 (Figure 4).

She was started on oral trametinib 1.5 mg and pazopanib 600 mg per day. A chest CT follow-up six months later showed stable right pleural thickening. However, the right pleural effusion continued to reoccur in a slower course, which was managed with monthly thoracentesis. Her clinical course was complicated by opioid-managed cancer-related chest pain, acneiform rash, and stomatitis attributed to trametinib managed with topical hydrocortisone.

## Discussion

The pleura is an infrequent primary site of EHE, with approximately 10% of cases having primary pleural involvement [4]. Its delayed presentation, difficulty establishing a tissue diagnosis, and aggressive nature make pleural EHE associated with the poorest prognosis compared to EHE involving other sites [3]. In addition to recurrent pleural effusions, it can present with unremitting chest pain, pleural thickening, hemoptysis if lung involvement exists, or lung entrapment due to severe pleural inflammation and scarring [5], as illustrated in our patient. The morphology of EHE can vary, but it is most often composed of epithelioid cells with dense or glossy eosinophilic cytoplasm organized in strands, cords, or nests. Large cells with rigid intracytoplasmic vacuoles suggestive of lumen formation (so called “blister cells”) may be present and include red blood cells, though this finding is not necessary for diagnosis [6]. Immunohistochemistry (IHC) of EHE consistently expresses markers of vascular differentiation, such as CD31, ERG, CD34, or FLI-1 [2]. Two pathognomonic genetic events are implicated in these tumors, which can also be highlighted on IHC: WWTR1:CAMTA1 fusion (t(1;3)(p36.3;q25) translocation, implicated in ∼90% of EHE cases, CAMTA-1 stain) or YAP1::TFE3 fusion (∼10% of cases, TFE3 stain) [5,7,8].

Surgical resection of pleural EHE is often not feasible due to advanced disease on presentation and the presence of distal metastasis. Given the rarity of EHE, systemic chemotherapy treatment for other sarcomas has been implemented in its management [9]. The CAMTA1-WWTR1 fusion leading to overexpression of the methyl ethyl ketone (MEK) signaling pathway is the driving oncogenic pathway for the development of EHE and has been a focus of interest for the use of targeted MEK inhibitors such as trametinib. A published phase II clinical trial investigated using trametinib 2 mg daily in 42 patients with EHE, 40% of patients had stable disease six months after starting treatment along with a reduction in cancer-related pain [10]. Pazopanib, an antiangiogenic targeted therapy against the VEGF signaling pathway, has shown long-term control of EHE growth in multiple case reports and retrospective analyses [11-13].

## Conclusions

Pleural EHE is a locally aggressive and under-reported vascular sarcoma. Video-assisted thoracoscopic (VATS) pleural biopsies are often warranted to confirm the diagnosis. To establish an optimal treatment protocol for this ultrarare neoplasm, randomized controlled clinical trials need to investigate molecular treatments targeting the WWTR1:CAMTA1 fusion pathway implicated in the carcinogenesis of EHE. 
